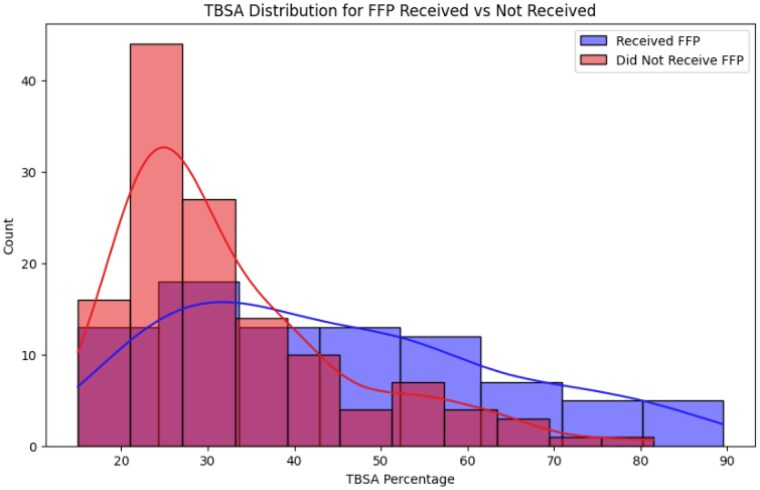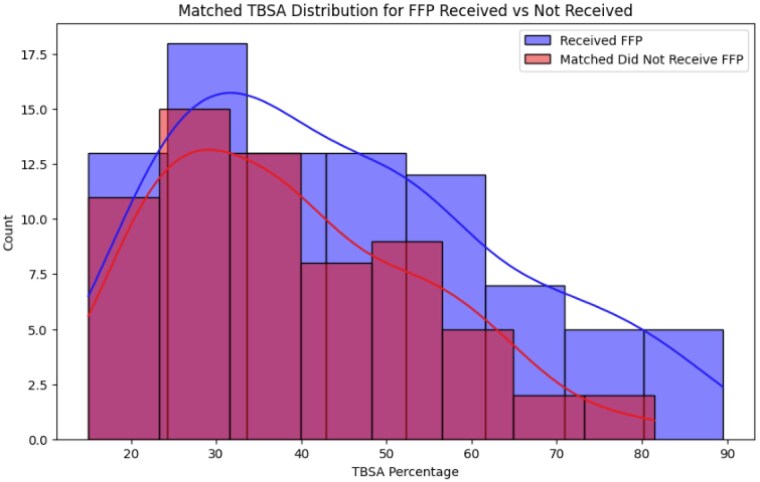# 86 Risk of Transfusion-Related Complications in Burn Patients Who Receive Fresh Frozen Plasma

**DOI:** 10.1093/jbcr/iraf019.086

**Published:** 2025-04-01

**Authors:** Kyle Mangum, krystal Morton, Michael Shalaby, Merry Mathew, Alan Pang, Rafael Cacao

**Affiliations:** Texas Tech University Health Sciences Center School of Medicine; Texas Tech University Health Sciences Center; Texas Tech University Health Sciences Center; Texas Tech University Health Sciences Center School of Medicine; Texas Tech University Health Sciences Center School of Medicine; Texas Tech University

## Abstract

**Introduction:**

Fresh frozen plasma (FFP) is a blood product commonly used during burn resuscitation, yet limited research exists regarding the risk of transfusion-related complications associated with FFP transfusion. Transfusion-related acute lung injury (TRALI) and transfusion-associated circulatory overload (TACO) are two complications that can significantly impact patient outcomes. It is expected to see an increase in transfusion related complication in patients who receive blood products, but how significant an impact this has is unclear. Understanding the risks, alongside the benefits, of FFP transfusion can inform clinical decision-making when managing resuscitation in burn patients.

**Methods:**

We conducted a retrospective cohort analysis to investigate the association between receiving FFP and the occurrence of TACO and TRALI in a matched patient cohort. The original dataset included 217 patients, of whom 86 received FFP and 131 did not. Key variables, such as the percentage of total body surface area burned (TBSA) and TACO/TRALI outcomes, were recorded. To address the imbalance in TBSA between the groups, we applied stratified sampling to match patients who received FFP with those who did not, resulting in 86 patients in the FFP group and 65 patients in the non-FFP group with similar TBSA distributions. The balance between the groups was verified using the Mann-Whitney U-test (p = 0.074). We then performed logistic regression analysis to investigate two hypotheses: (1) whether receiving FFP was associated with the occurrence of TACO and TRALI, and (2) whether the amount of FFP received was correlated with these outcomes.

**Results:**

Logistic regression indicated that receiving FFP was significantly associated with an increased likelihood of TRALI (OR = 5.11, p = 0.037) but not TACO (p = 0.436). However, when analyzing the amount of FFP administered, no statistically significant association was found with either TACO (p = 0.517) or TRALI (p = 0.328).

**Conclusions:**

It is expected to see an increase in TRALI for any patient population receiving blood products. However, these findings suggest that while receiving FFP may increase the likelihood of TRALI, the actual volume of FFP administered does not appear to play a significant role in the development of either TACO or TRALI.

**Applicability of Research to Practice:**

This study helps clarify the risks of FFP transfusion in burn patients and highlights directions for future research on this topic.

**Funding for the Study:**

N/A